# Stretchable zein-coated alginate fiber for aligning muscle cells to artificially produce cultivated meat

**DOI:** 10.1038/s41538-024-00257-y

**Published:** 2024-02-19

**Authors:** Dayi Jeong, Goo Jang, Woo Kyung Jung, Yong Ho Park, Hojae Bae

**Affiliations:** 1 Department of Stem Cell and Regenerative Biotechnology, KU Convergence Science and Technology Institute, Konkuk University, 120 Neungdong-ro, Gwangjin-gu, Seoul, 05029 Republic of Korea; 2 Laboratory of Theriogenology and Biotechnology, Department of Veterinary Clinical Science, College of Medicine and the Research Institute of Veterinary Science, Seoul National University, Seoul, 08826 Republic of Korea; 3 NoAH Biotech Co., Ltd., Suwon-si, Gyeonggi-do 16614 Republic of Korea; 4 Department of Microbiology, College of Veterinary Medicine and Research Institute for Veterinary Science, Seoul National University, 1 Gwanak-ro, Gwanak-gu, Seoul, 08826 Republic of Korea; 5 Institute of Advanced Regenerative Science, Konkuk University, 120 Neungdong-ro, Gwangjin-gu, Seoul, 05029 Republic of Korea

**Keywords:** Biomaterials - cells, Tissues

## Abstract

Numerous studies have explored the cultivation of muscle cells using non-animal materials for cultivated meat production. Achieving muscle cell proliferation and alignment using 3D scaffolds made from plant-based materials remains challenging. This study introduces a technique to culture and align muscle cells using only plant-based materials, avoiding toxic chemical modifications. Zein-alginate fibers (ZA fibers) were fabricated by coating zein protein onto alginate fibers (A fibers). Zein’s excellent cell compatibility and biodegradability enable high cell adhesion and proliferation rates, and the good ductility of the ZA fibers enable a high strain rate (>75%). We demonstrate mature and aligned myotube formation in ZA fibers, providing a simple way to align muscle cells using plant-based materials. Additionally, cultivated meat was constructed by assembling muscle, fat, and vessel fibers. This method holds promise for the future mass production of cultivated meat.

## Introduction

The production of cultivated meat using tissue engineering technology and cells obtained from livestock has undergone rapid development in recent years^1^. The need for alternative protein sources to replace real meat, given concerns over animal welfare and environmental problems arising from factory farming, as well as the prospect of global meat consumption rising to 73% by 2050, has been the driving force behind the development of cultivated meat^2^. Unlike plant-based meat analogs, cultivated meat is made of tissues originating from livestock cells that have been cultivated in large quantities, making its taste and nutritional composition more similar to that of real meat. Furthermore, cultivated meat can provide a safe and sustainable alternative to animal protein because, unlike traditional meat, it is produced in a controlled and sterile environment^3,4^. To produce cultivated meat, skeletal muscle satellite cells, such as bovine satellite cells (BSCs)^5^ or pig satellite cells^6,7^, are attached, multiplied, and differentiated into skeletal muscles in a 3D scaffold, which provides a supporting matrix^8^. The 3D scaffold should have good biocompatibility, mimic the natural microenvironment of the extracellular matrix (ECM), and provide appropriate structural and mechanical properties^9^. 3D scaffolds have also been utilized to align muscle cells to simulate actual muscle tissue^10–12^. However, various biocompatible scaffolding biomaterials, such as porous scaffolds, fiber scaffolds, and hydrogels, are generally made from synthetic or animal-derived biopolymers^13^. In addition, muscle cell alignment methods, which are typically suited for aligning one or a small number of strands^14^, present difficulties in the context of cultivated meat, where mass production is required.

Recently, plant-derived proteins have become increasingly recognized as promising natural biopolymers owing to their non-animal origin, low cost, satisfactory biocompatibility, biodegradability, and structural diversity^15,16^. Ben-Arye et al. demonstrated the growth and maturation of BSCs in porous soy protein scaffolds, which are scalable and inexpensive^17^. Wheat gluten and arginine-glycine-aspartate-modified alginate mixed with pea protein isolate^18^ and soy protein isolate^19^ have also been used as raw materials in porous scaffold construction.

Zein, a corn prolamine protein, is considered a safe food ingredient by the Food and Drug Administration (FDA) and can be obtained as a by-product of the corn processing industry^20^. In addition, owing to its high flexibility, biocompatibility, and biodegradability, it has gained significant attention in the biodegradable film coating, food, and pharmaceutical industries^21,22^. When combined with synthetic biopolymers, zein shows promise in enhancing the mechanical stiffness and cell affinity of scaffolds in tissue engineering^23^. However, the poor rheological, electrical, and hydrophobic properties of pure zein proteins hinder their application in the manufacturing of edible scaffolds^24^.

**Fig. 1:**
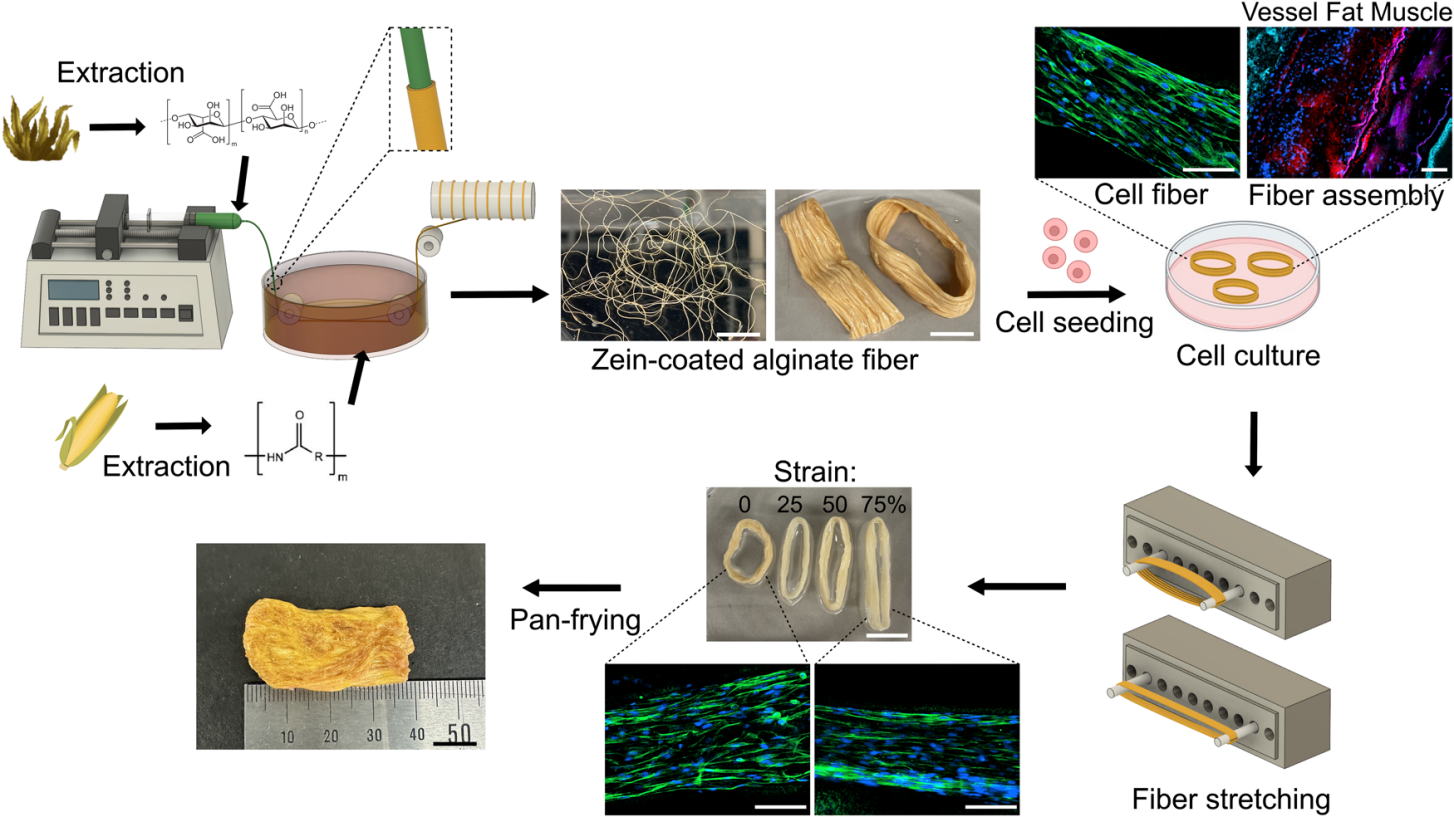
Schematic representation of the development of a zein-coated alginate (ZA) fiber scaffold for cultivated meat production using a wet-spinning technique. Alginate was injected into a zein coagulation bath to form ZA fibers. Bovine satellite cells, adipocytes, and an endothelial cell line were then seeded onto the fiber to mimic vessel, fat, and muscle tissue, and allowed to proliferate. The fibers were stretched using a three-dimensional printed stretching device to stimulate muscle formation and align cells. The resulting meat was pan-fried, and the texture resembled that of animal meat. Scale bar = 1 cm for photographs of fiber scaffolds and 100 µm for fluorescence microscopy images.

To overcome these limitations, we present the development of a fiber scaffold with high cell affinity and ductility by leveraging the hydrophobic nature of zein and the hydrophilic properties of sodium alginate. Sodium alginate, a natural hydrophilic polysaccharide extracted from brown algae, exhibits exceptional molding and mechanical properties^25^. It is widely used in the fabrication of hydrogel fibers using wet-spinning techniques due to its rapid cross-linking ability in the presence of calcium ions^26^. However, cell proliferation and differentiation within alginate hydrogels are limited because cells lack receptors that recognize alginate^27^. Therefore, in this study, a zein-coated alginate fiber (ZA fiber), consisting of a zein shell surrounding an alginate fiber (A fiber), was developed using a wet-spinning technique (Fig. 1). Hydrophilic alginate was injected into a coagulation bath of a zein solution containing calcium ions, resulting in the formation of alginate fibers. The hydrophobic zein shell formed outside the alginate fiber increased the cell affinity on the fibers. We optimized the fabrication process by evaluating the effects of zein concentration and fiber diameter on the mechanical properties of the resulting scaffold and characterized the composition of fibers using scanning electron microscopy (SEM). SEM was employed to characterize the internal A fiber and external zein-coated shell surrounding the A fiber. We then investigated cell viability and adherence in the ZA fibers, as well as the morphological changes in cells in response to diameter variations in the fibers. Additionally, we assessed cell alignment and the cellular response to physical stretch stimulation using a stretching device developed in the laboratory. We demonstrated a straightforward, rapid, and effective method for aligning muscle cells by stretching the bundle of ZA fibers after cultivating the cells within the bundle. Finally, we developed an innovative approach for cultivated meat production using the newly developed scaffold to culture and assemble muscle, fat, and vessel cells, effectively mimicking real meat.

## Results

### Fabrication of continuous zein-coated alginate fiber

**Fig. 2:**
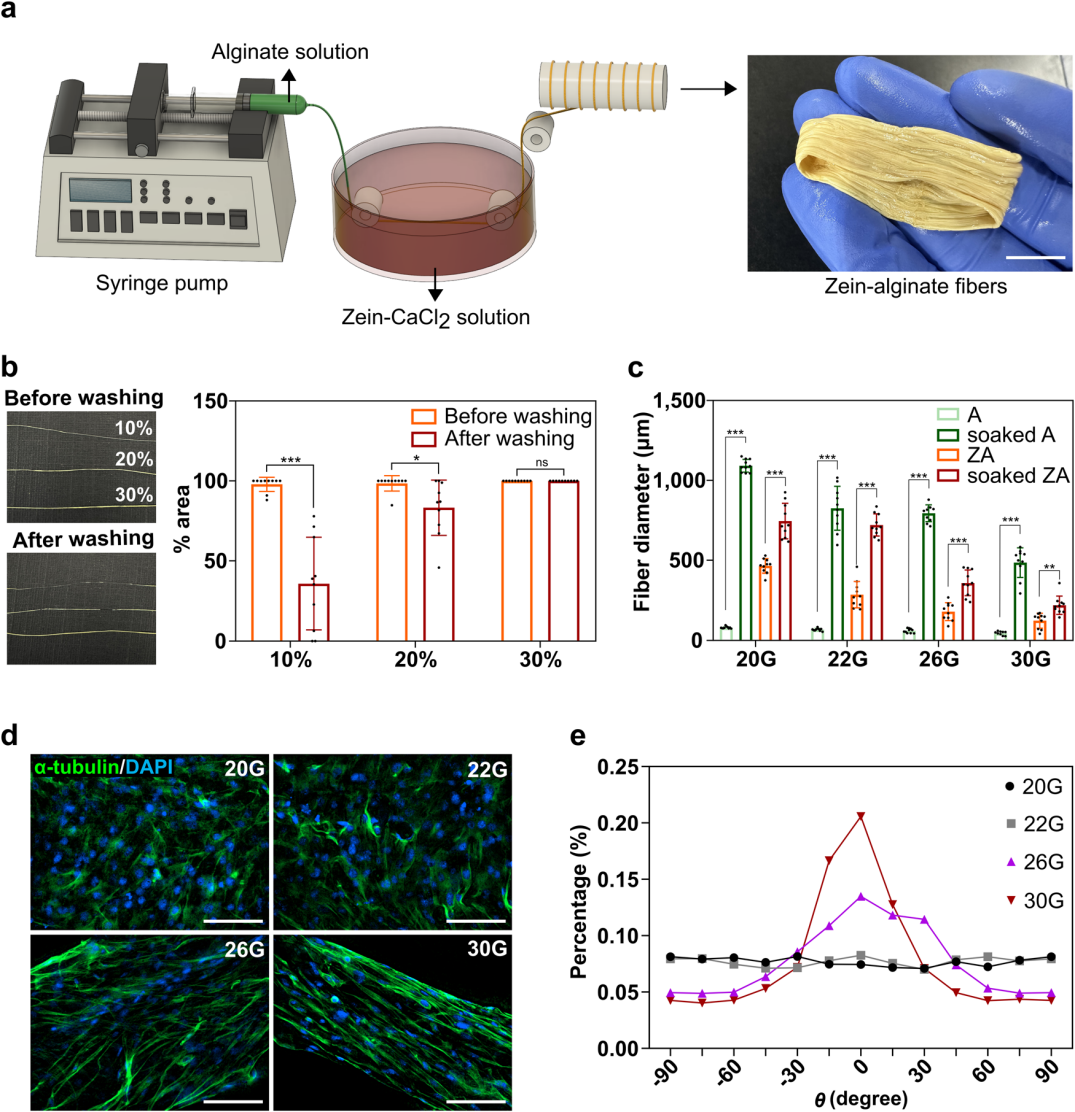
Optimization of fiber fabrication. **a** Illustration of the preparation of zein-coated alginate (ZA) fibers using a wet-spinning technique. Scale bar = 1 cm. **b** Photograph of ZA fibers with different zein concentrations (10%, 20%, 30%) and quantification of the % area of remaining zein after phosphate-buffered saline washing in each group. Data are shown as the means ± standard deviation (s.d.) (*n* = 10; *P < 0.05; ***P < 0.001; ns, not significant, according to Student’s *t* test). **c** Relationship between fiber diameter and needle diameter in four groups (alginate (A), soaked A, ZA, and soaked ZA fibers). Data are presented as means ± standard deviation (s.d.) (*n* = 10; **P < 0.01; ***P < 0.001) and were analyzed using one-way ANOVA followed by Tukey’s post-hoc test. **d** Fluorescence microscopy images of C2C12 cells treated with ZA fibers produced using different needle diameters (20 G, 22 G, 26 G, and 30 G). Cells were immunostained with α-tubulin (green), and nuclei were stained with 4,6-diamidino-2-phenylindole dihydrochloride (DAPI; blue). Scale bar = 100 µm. **e** Alignment analysis of C2C12 cells within ZA fibers produced using different needle diameter sizes (20 G, 22 G, 26 G, and 30 G). Cells with an alignment angle close to 0° indicate that they are oriented along the microfibers.

To optimize the fabrication of ZA fibers, using a wet-spinning technique, alginate was injected into a zein coagulation bath containing CaCl_2_ to form A fibers coated with a zein shell (Fig. 2a). The A fibers solidified within the coagulation bath, allowing for direct contact with zein. This facilitated the adhesion of zein onto the fiber surfaces, leading to the formation of a zein shell or coating. Finally, the fibers, now coated with the zein shell, were collected from the coagulation bath. We evaluated the effects of zein concentration and fiber diameter on the resulting structure. The zein coating shell on the surface of the A fiber is formed through hydrophobic interactions without the addition of a chemical cross-linking agent. Therefore, we hypothesized that the zein coating is susceptible to collapse under external stimulation, particularly at low zein concentrations. We determined the optimal zein concentration required to prevent peeling or breaking of the zein shell. Alginate (1%) was injected into a zein coagulation bath containing zein concentrations of 10, 20, and 30%. The remaining zein shell area was measured after washing with phosphate-buffered saline (PBS) (Fig. 2b). The results showed that 62% and 15% of the zein shells were lost after washing at zein concentrations of 10% and 20%, respectively. In contrast, at a zein concentration of 30%, 100% of the zein shells were preserved even after washing. Therefore, we established that the solidified zein-coated shell is unable to maintain a stable shape below a zein concentration of 30%, and thus, subsequent experiments were performed using a fixed zein concentration of 30%.

Considering the importance of microscale three-dimensional (3D) fiber diameters in cell orientation^28^, we investigated the minimum fiber diameter by reducing the needle diameter from 20 G to 30 G (Fig. 2c). The produced A fibers and ZA fibers decreased in size with a reduction in needle diameter. Examining the dry and soaked fibers cultured for 48 h, we observed that dry A fibers (20 G, 22 G, 26 G, and 30 G needles) were all under 100 μm, with soaked diameters increasing approximately 12.3-fold. Dry ZA fibers (20 G, 22 G, 26 G, and 30 G needles) exceeded 100 μm, but their swelling ratio after soaking was notably lower than that of A fibers, resulting in smaller diameters. These results demonstrate that the zein shell acts as a physical barrier to limit alginate swelling, thereby reducing fiber swelling and resulting in a smaller diameter than that of A fibers.

To investigate the morphological changes in cells in response to diameter variations in ZA fibers, we conducted immunofluorescence staining 6 days after C2C12 (a mouse embryonic myoblast cell line) attachment to assess cell orientation. Fluorescence images revealed that the cell orientation along the microfibers became more pronounced as the fiber diameter decreased (Fig. 2d). Additionally, we investigated cell alignment and found that cells became more aligned as the fiber diameter decreased (Fig. 2e).

We successfully produced ZA fibers with aligned cells using a 30 G needle and a 30% zein concentration. The dry fibers had an average diameter of approximately 124 μm, whereas the soaked fibers had an average diameter of approximately 220 μm.

### Characterization of zein-coated alginate fiber

**Fig. 3:**
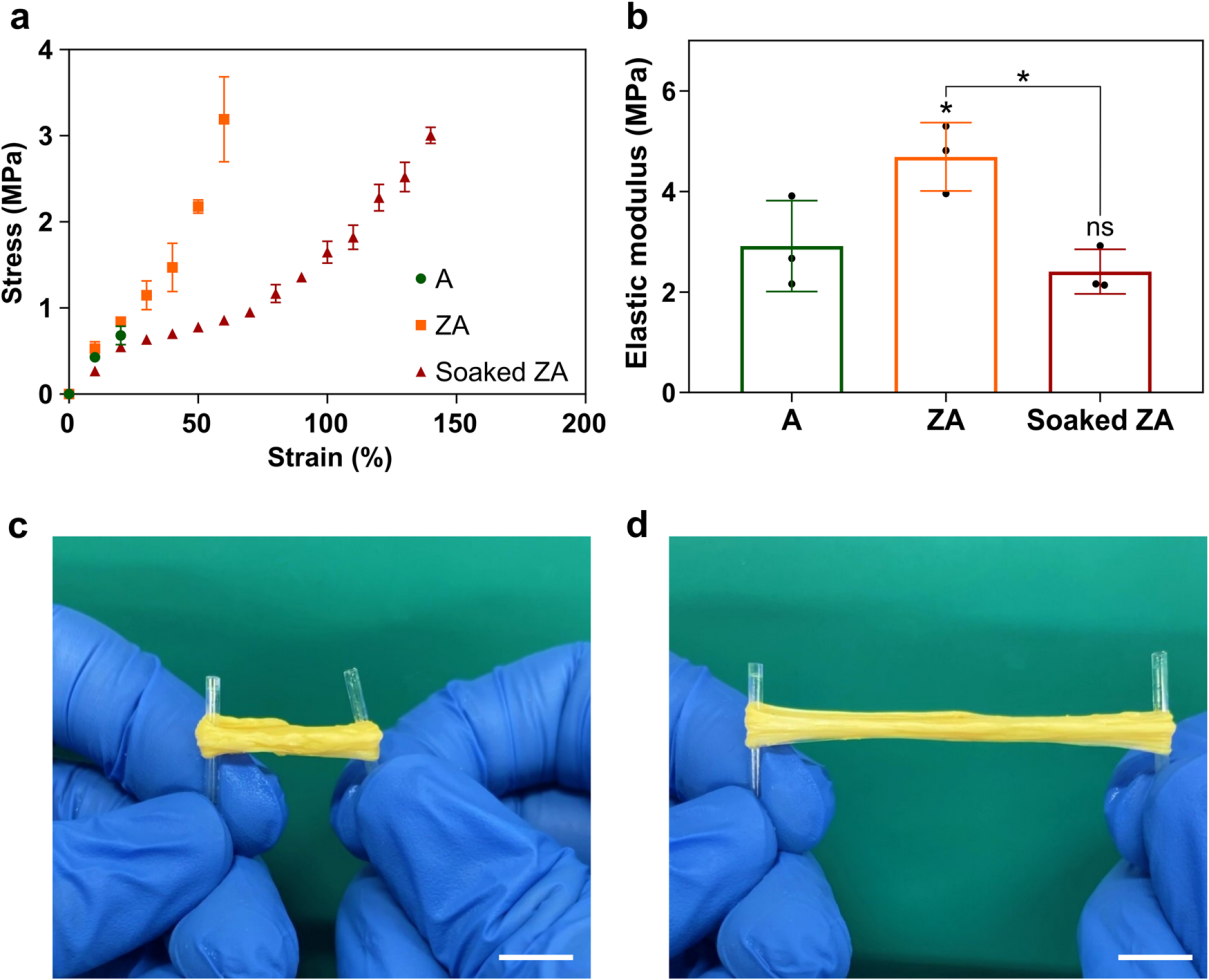
Mechanical properties of fibers. **a** Tensile stress-strain curve of alginate (A), zein-coated alginate (ZA), and soaked ZA fibers. Data are shown as the means ± standard deviation (s.d.) (*n* = 3). **b** Calculated elastic modulus of A, ZA, and soaked ZA fibers. Data are presented as means ± standard deviation (s.d.) (*n* = 3; *P < 0.05; ns, not significant) and were analyzed using one-way ANOVA followed by Tukey’s post-hoc test. Photograph of the ZA fiber scaffold bundle before (**c**) and after stretching (**d**). Scale bar = 1 cm.

Tensile tests were conducted to evaluate the mechanical properties of the ZA fibers, and the stress-strain curves of the four groups (A fiber and ZA fiber 1 h after production, soaked A fiber and soaked ZA fiber immersed in PBS for 48 h after production) were plotted (Fig. 3a, b). The dry A fiber exhibited tensile stress at a break of 0.68 MPa, an elastic modulus of 3.04 MPa, and the tensile strain at break was less than 25%. In contrast, the dry ZA fiber showed significantly higher values with tensile stress at a break of 4.61 MPa, an elastic modulus of 4.63 MPa, and tensile strain at a break of more than 60%. Measurements for the soaked A fiber were impossible because of its poor elasticity and inability to withstand high strain ratios. The soaked ZA fiber exhibited tensile stress at a break and elastic modulus of 2.79 MPa and 2.54 MPa, respectively, which were lower than those of the ZA fiber. However, its tensile strain at break was significantly increased to more than 140%. Notably, the soaked ZA fiber bundle could be highly stretched without breaking owing to its high ductility (Fig. 3c, d, and Supplementary material: Video 1). These results suggest that the ductility of zein-coated shells is significantly improved in the presence of moisture.

**Fig. 4:**
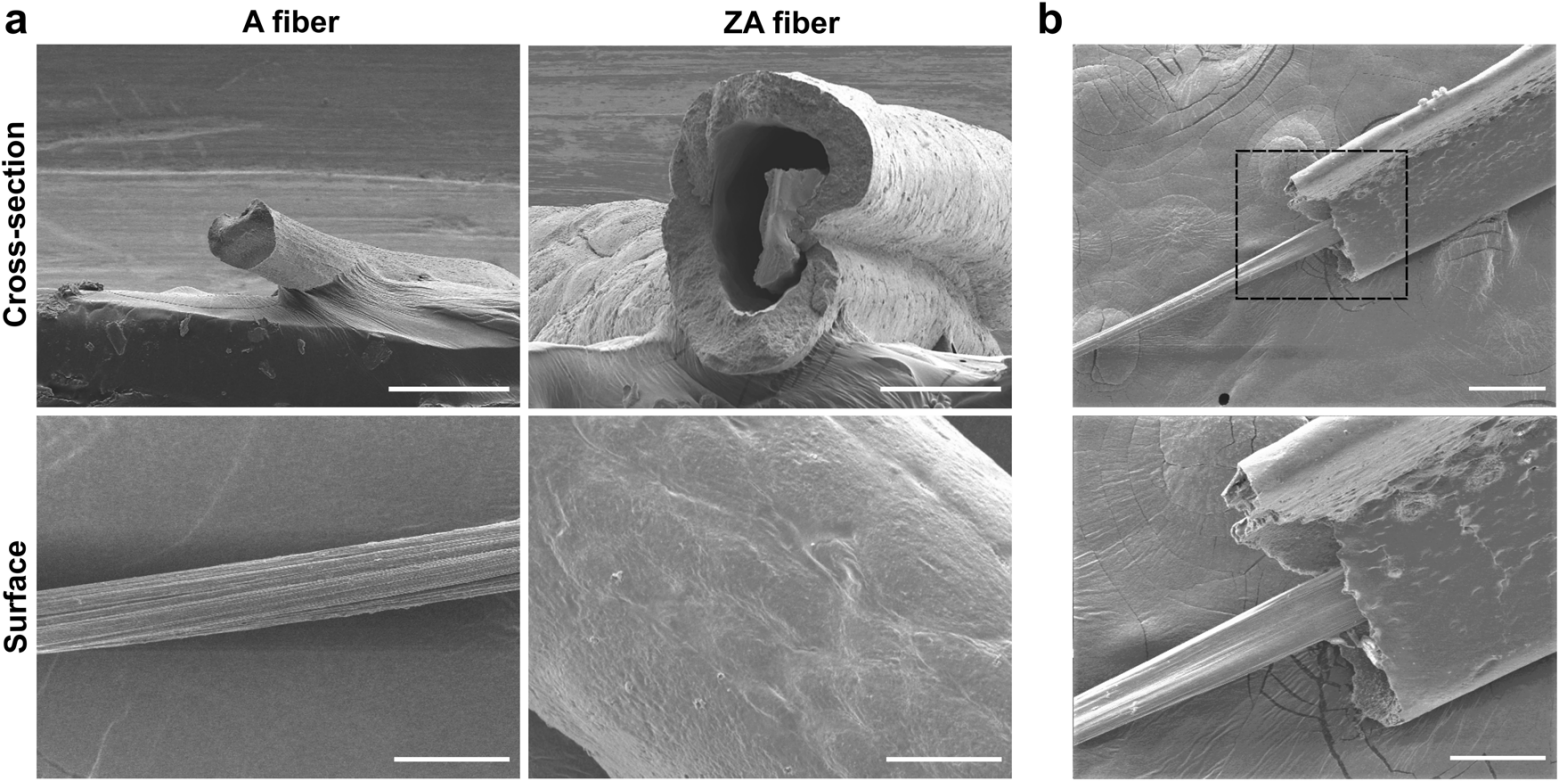
Characterization of fiber composition using scanning electron microscopy (SEM). **a** SEM images of cross-section and surface of alginate (A) and zein-coated alginate (ZA) fibers. Scale bars = 100 and 50 μm for cross-section and surface images, respectively. **b** SEM image of internal alginate fiber and zein-coated shell. Scale bars = 100 μm (cross-sections) and 50 μm (surface).

SEM images of the A and ZA fibers (Fig. 4a, b) confirmed the presence of internal A fibers and external zein-coated shells. Furthermore, an empty space between the alginate fiber and the zein-coated shell was observed when the moisture of the alginate in the ZA fiber was drained, which is consistent with the results shown in Fig. 2b, indicating that the zein-coated shell can limit the swelling of the alginate fiber owing to its hydrophobic physical barrier.

### Cell-fiber interaction (proliferation)

**Fig. 5:**
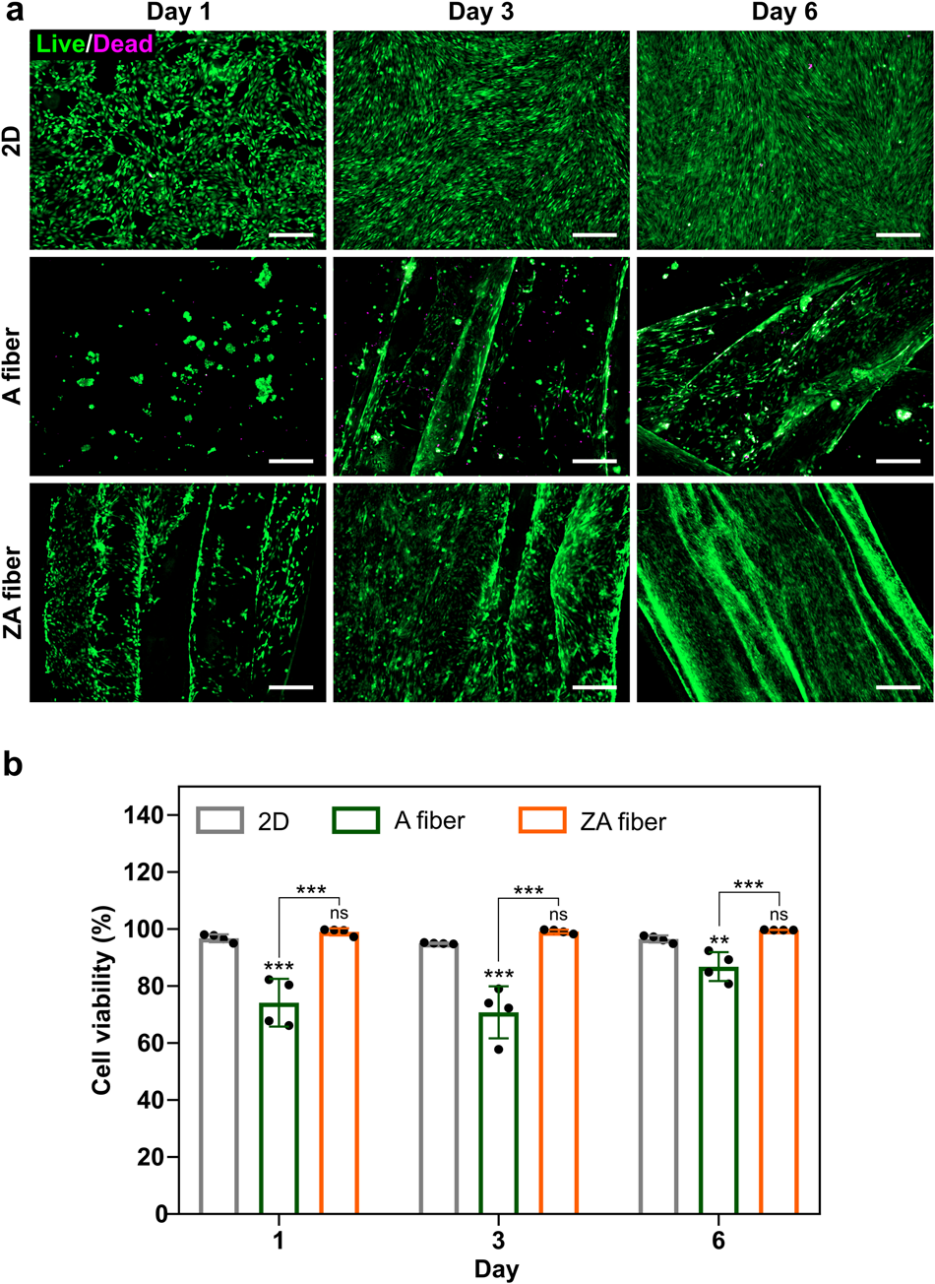
Cell viability using different culture methods. **a** Live (green)/dead (magenta) staining assay of C2C12 cells grown in a 2D culture plate, alginate (A) fiber, and zein-coated alginate (ZA) fiber for 1, 3, and 6 days. Scale bars = 300 μm. **b** Quantification of cell viability obtained from live/dead assays at 1, 3, and 6 days. Data are presented as means ± standard deviation (s.d.) (*n* = 4; **P < 0.01; ***P < 0.001; ns, not significant) and were analyzed using one-way ANOVA followed by Tukey’s post-hoc test.

To investigate cell viability and adherence in the ZA fibers, C2C12 cells were seeded onto the fiber, after which their proliferation was observed up to day 6 and compared with that in the two-dimensional (2D) environment and A fibers. Alginate is known to have limited cell attachment motifs^27^; hence, cells tend to clump and exhibit limited attachment to A fibers. In contrast, the ZA fibers exhibited high cell adhesion ability and enabled proliferation without clumping (Fig. 5a). Quantitative analysis of cell viability showed that cell viability was higher in the ZA fibers than in the A fibers, indicating that zein provided an appropriate environment for cell attachment and proliferation (Fig. 5b).

### Fiber stretching for aligned myotubes

Various studies have aimed to simulate the alignment of muscle cells to mimic in vivo muscle structure. One way to achieve muscle simulation is to align muscle cells in a single direction using methods such as electrical stimulation or stretching^29,30^. In this study, a bundle of muscle fibers was stimulated by winding highly ductile ZA fibers into bundles and performing physical stretching. To investigate the cellular response to physical stretch stimulation, a stretching device capable of uniaxial stretching was developed using Digital Light Processing (DLP) 3D printing with an acrylated epoxidized soybean oil/polyethylene glycol diacrylate (AESO/PEGDA) bioink.

**Fig. 6:**
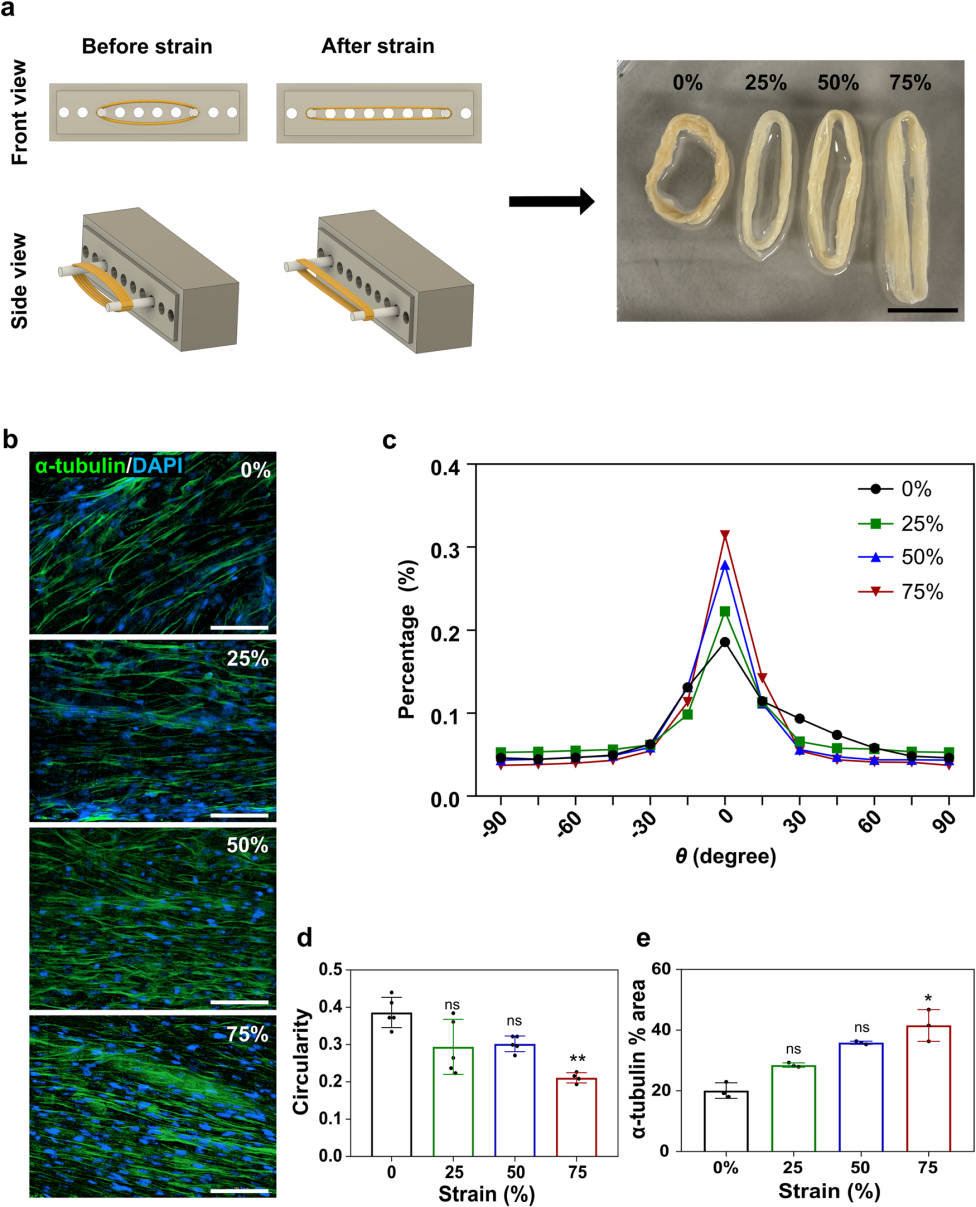
Effect of straining on cell morphology and alignment. **a** Schematic illustration of physical stretch stimulation of zein-coated alginate (ZA) fibers using a 3D-printed stretching device. Right: photograph of ZA fibers after applying different strain ratios (0, 25, 50, and 75%). Scale bar = 1 cm. **b** Fluorescence microscopy images of C2C12 cells in ZA fiber after applying different strain ratios (0, 25, 50, and 75%). Cells were immunostained with α-tubulin (green) and nuclei were stained with 4,6-diamidino-2-phenylindole dihydrochloride (DAPI) (blue). Scale bar = 100 µm. **c** Alignment analysis of C2C12 cells within ZA fiber after applying different strain ratios (0, 25, 50, and 75%). Cells with an alignment angle close to 0° indicate that they are oriented along the microfiber. **d** Circularity of nuclei and (**e**) α-tubulin % area were measured using data from immunofluorescence images. Data are presented as means ± standard deviation (s.d.) (*n* = 5 for (**d**) and *n* = 3 for (**e**); *P < 0.05; **P < 0.01; ns, not significant) and were analyzed using one-way ANOVA followed by Tukey’s post-hoc test.

The AESO/PEGDA bioink is commonly used as an eco-friendly bioresin alternative in 3D printing because of its capacity to enhance mechanical properties and enable rapid and sophisticated photo-crosslinking^31–34^. The strain ratio was adjusted by placing a bundle of ZA fibers in the holder of the stretching device and moving the holder (Fig. 6a). Although ZA fibers can withstand high strain ratios (more than 140%), we applied strain ratios of 0, 25, 50, and 75% because animal skeletal muscle achieves a 40% strain ratio during contraction^35^. C2C12 cells were incubated in a bundle of ZA fiber wound to a 0% strain ratio on the stretching device for 5 days, prior to being transferred to the strain device. The strain ratio was increased to 0, 25, 50, and 75%, and cells were incubated for an additional day. By examining the morphology of cells subjected to different strains using immunofluorescence staining, we confirmed that the cells became denser and more uniformly oriented as the strain increased (Fig. 6b, c). Furthermore, fluorescence image analysis showed that the circularity of the nuclei decreased as the cells aligned (Fig. 6d) and became more densely concentrated (Fig. 6e). These results demonstrate that muscle cells can be more tightly aligned in a single direction, similar to actual muscle fibers, by culturing and stretching them in flexible ZA fibers. Previous studies have shown limitations in stretching techniques for the mass production of cultivated meat, as they were capable of stretching only individual strands or a small number of strands^14^. Therefore, this study holds significant relevance as it introduces a method that enables the simultaneous stretching of multiple strands, potentially facilitating the mass production of cultivated meat.

### Myogenesis on zein-coated alginate fibers

**Fig. 7:**
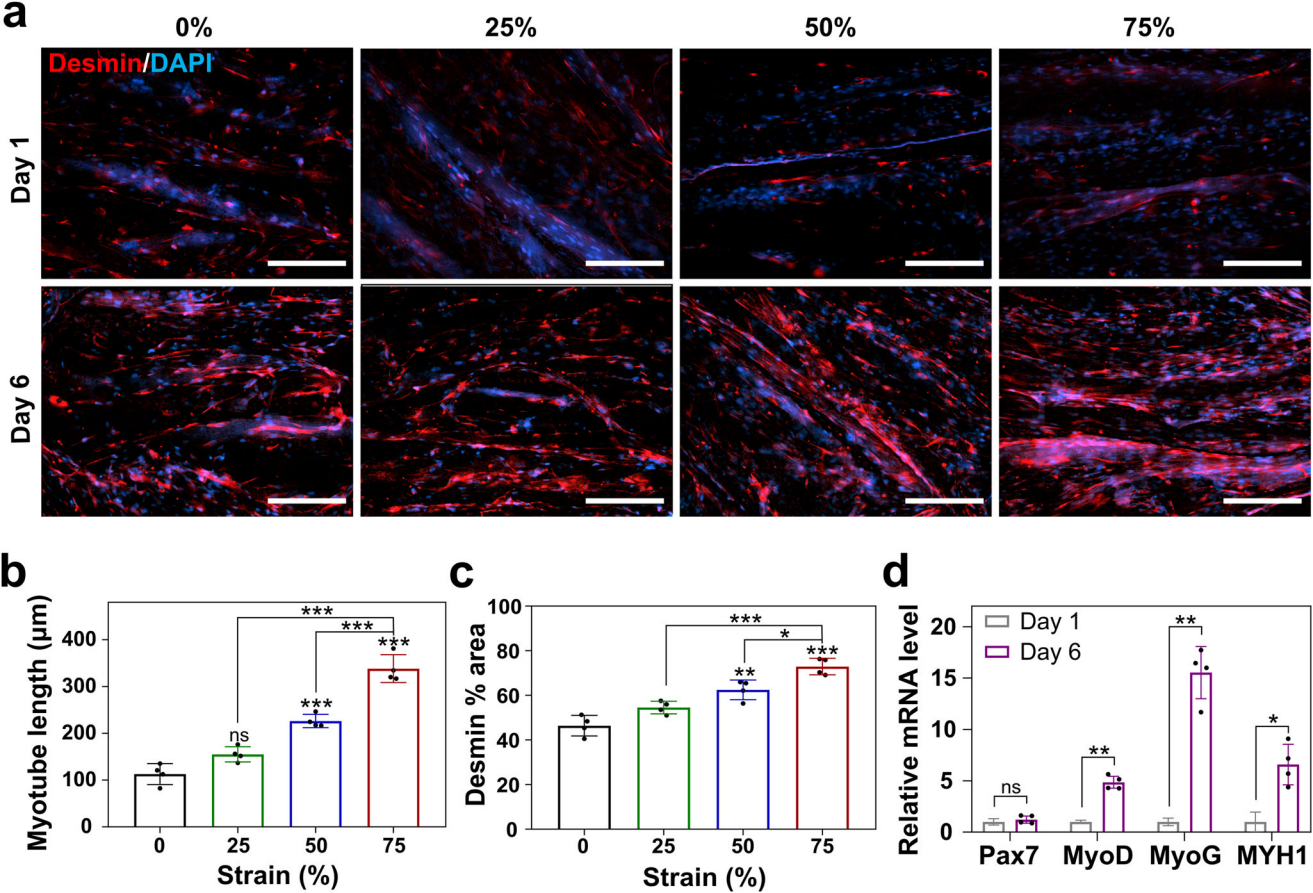
Effect of straining on myotube differentiation and maturation. **a** Fluorescence microscopy images of C2C12 cells after myogenic differentiation in zein-coated alginate (ZA) fibers subjected to different strain ratios (0, 25, 50, and 75%). Cells were immunostained for desmin (red), and nuclei were stained with 4,6-diamidino-2-phenylindole dihydrochloride (blue). Scale bar = 200 µm. **b** Myotube length and (**c**) desmin % area were measured using data from immunofluorescence images. Data are presented as means ± standard deviation (s.d.) (*n* = 4; *P < 0.05; **P < 0.01; ***P < 0.001; ns, not significant) and were analyzed using one-way ANOVA followed by Tukey’s post-hoc test. **d** RT-qPCR analysis of C2C12 cells in ZA fibers subjected to a strain ratio of 75% 1 and 6 days after differentiation. Data are presented as means ± standard deviation (s.d.) (*n* = 4; *P < 0.05; **P < 0.01; ns, not significant, calculated using Student’s *t* test).

C2C12 myoblasts were cultured in ZA fibers under varying strain ratios (0, 25, 50, and 75%) to investigate their impact on myotube development. After 1 and 6 days of differentiation, immunostaining for desmin, a myogenic marker expressed during myotube maturation^36^, was performed (Fig. 7a). Fluorescence images showed that desmin expression increased from day 1 to 6, and the myotubes became longer and denser with increasing strain ratios. Quantitative analysis of the fluorescence images demonstrated that myotube length correlated with the strain ratio (113 ± 19 μm, 155 ± 14 μm, 229 ± 12 μm, and 344 ± 27 μm for 0, 25, 50, and 75% strain ratios, respectively) (Fig. 7b). Furthermore, the percentage of desmin-positive cells increased with higher strain ratios (46, 55, 63, and 75%) (Fig. 7c). Analysis of the RNA levels of myogenic genes in C2C12 cells in ZA fibers subjected to a 75% strain ratio showed a minor increase in expression of *Pax7* on day 6 compared with that on day 1. Additionally, *MyoD*, *MyoG*, and *MYH1* expression showed approximately 4.8-, 15.5-, and 6.7-fold increases, respectively, from day 1 to day 6 (Fig. 7d). This result is consistent with the fact that expression of *Pax7* initially increases and then decreases during muscle cell myogenesis, whereas *MyoD*, *MyoG*, and *MYH1* are highly expressed^37^. These results suggest that physical strain applied to ZA fibers can promote the differentiation, maturation, and elongation of muscle cells.

### Muscle, fat, and vessel tissue fabrication

**Fig. 8:**
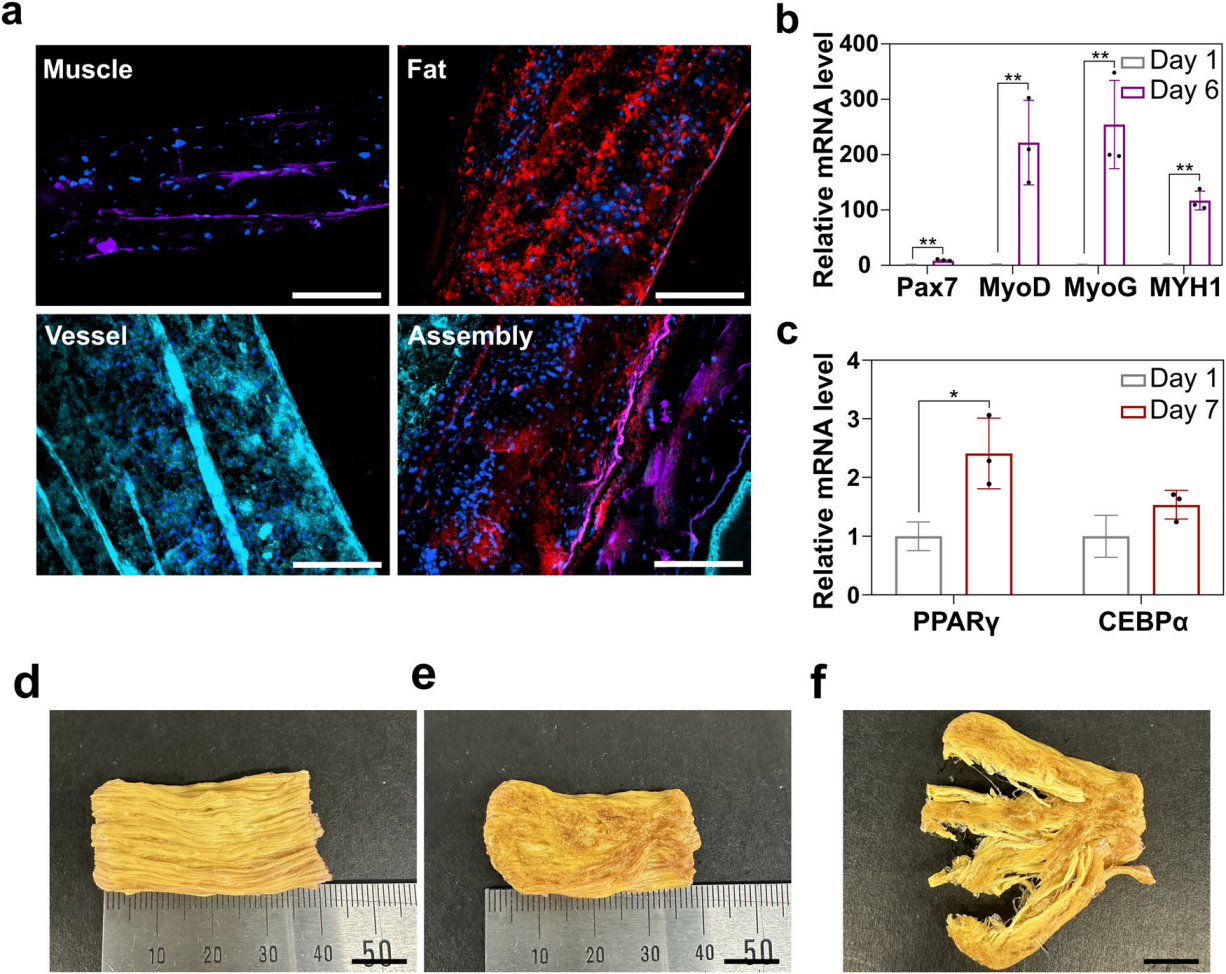
Culture and assembly of muscle, fat, and vessel fibers to create artificial meat. **a** Fluorescence microscopy images of muscle fibers (myotubes from bovine satellite cells; desmin, purple), fat fibers (adipocytes from primary adipocytes; Nile red, red), and vessel fibers (CPAE cell line; VE-cadherin, cyan blue). Nuclei were stained with 4,6-diamidino-2-phenylindole dihydrochloride (blue). Scale bar = 200 µm. **b** RT-qPCR analysis of primary myoblasts 1 and 6 days after differentiation. Data are shown as means ± standard deviation (s.d.) (*n* = 3; **P < 0.01, calculated using Student’s *t* test). **c** RT-qPCR analysis of primary adipocytes 1 and 7 days after differentiation. Data are shown as means ± standard deviation (s.d.) (*n* = 3; *P < 0.05, calculated using Student’s *t* test). **d** Photograph of ZA fiber-cultivated meat prior to pan-frying and (**e**) after pan-frying. Scale bar = 1 cm. **f** Photograph of pan-fried cultivated meat pulled apart. Scale bar = 1 cm.

In addition to muscle tissue, meat contains fat, blood vessels, and ECM^3^. In previous studies, attempts have been made to produce cultivated meat by assembling muscle, fat, and vessel cells using the tendon-gel integrated bioprinting technique to mimic the actual composition of meat^38^. However, this method uses collagen, an animal-derived material, which limits its application in the mass production of cultivated meat. To address this issue, ZA fibers, a plant-based material, were used to culture and assemble muscle, fat, and vessel cells to create cultivated meat. To produce muscle fibers, primary BSCs were cultured and differentiated into ZA fibers. Aligned myotubes of BSCs were observed in the immunostaining images (Fig. 8a, Muscle), and RNA analysis confirmed the high expression levels of *MyoD*, *MyoG*, and *MYH1* (Fig. 8b). For fat fibers, primary bovine preadipocytes were cultured and differentiated into adipocytes using linoleic acid (LA) instead of conventional drugs to create edible cultivated meat^39,40^. Nile red staining demonstrated the presence of lipid droplets within the fibers (Fig. 8a, Fat), and RNA analysis confirmed high expression of *PPARγ* and *CEBPα* during adipocyte differentiation (Fig. 8c). Vessel fibers expressing VE-cadherin were also obtained by culturing CPAE cells, a bovine endothelial cell line (Fig. 8a, Vessel). Finally, the muscle, fat, and vessel fibers were successfully assembled to create the three components of meat (Fig. 8a, Assembly).

To produce cultivated meat, primary myoblasts were cultivated in ZA fiber scaffolds for 10 days, and the resulting cultivated meat was pan-fried in a preheated cast iron pan. The pan-fried meat was similar in size to the non-pan-fried meat and had a yellow-brown color after pan-frying (Fig. 8d, e, Supplementary material: Video 2). Notably, upon pulling the pan-fried meat apart, it was observed that the fiber structure remained intact, giving rise a desirable fibrous texture (Fig. 8f).

This study demonstrated the successful production and assembly of muscle, fat, and vessel fibers using plant-based materials, specifically zein and alginate. These findings offer significant advantages in the scalability and mass production of cultivated meat. By culturing the muscle, fat, and vessel fibers separately before assembling them in the desired proportions and shapes, this approach has potential for future applications in meat culturing.

## Discussion

The minimization of animal-derived materials is crucial for achieving sustainable production of cell-cultivated meat. In this study, we demonstrated a scaffold for cultivated meat using zein protein and alginate derived from natural plant materials: corn and brown algae. Zein protein is insoluble in water owing to its hydrophobic properties^41^, making its application in tissue engineering difficult despite its high biocompatibility^24,42^. We found that when the zein solution encounters the hydrophilic alginate hydrogel, its solubility decreases and it solidifies on the alginate surface. Therefore, we utilized these properties to produce zein-coated alginate fibers using a wet-spinning technique; the resulting fibers exhibited high elasticity, enabling muscle alignment. Furthermore, the assembly of muscle, fat, and vascular cells on these fibers demonstrate the potential to mimic the major components of meat.

Zein enhanced the cytocompatibility and structural stability of alginate hydrogel, enabling the creation of the desired texture and structure in cultivated meat. The wet spinning technique used to fabricate the fibers and the optimization of zein concentration and fiber diameter allowed precise control over the scaffold properties (Fig. 2). Specifically, a 30% zein concentration was identified as optimal for preserving the zein coating around alginate fibers, preventing it from peeling or breaking. Moreover, reducing the needle diameter during fiber production played a crucial role in achieving smaller diameters for ZA fibers, influencing cell alignment and direction that are important for tissue engineering^28^. The observed lower swelling rate for ZA fibers provides mechanistic insight into fiber-cell interactions attributable to the zein coating. This restriction on alginate fiber swelling implies enhanced structural stability, mechanistically linked to improved cell adhesion, proliferation, and long-term viability.

The mechanical significance of zein-coated shells is further emphasized in promoting the robustness of the scaffold in the tensile test (Fig. 3). ZA fibers exhibit higher tensile stress at break, elastic modulus, and tensile strain at break than A fibers, demonstrating the mechanical advantages of the zein-coated shell. Additionally, soaked ZA fibers demonstrated excellent ductility while maintaining high strain levels, making them particularly suitable for applications requiring mechanical flexibility^43^. Scanning electron microscopy (SEM) images confirmed the presence of internal A fibers and external zein coating shells (Fig. 4), supporting the structural integrity and barrier function of the zein coating.

The interaction between cells and ZA fibers is crucial for the research on cultivated meat, where large-scale cell attachment and cultivation are essential^1,44^. Cultivating C2C12 cells on ZA fibers significantly enhanced cell viability and adhesion compared to their cultivation on A fibers (Fig. 5). The restricted cell attachment motif of alginate led to aggregation on A fibers^27^, reducing cell adhesion, while ZA fibers promoted cell adhesion and proliferation without aggregation. These results demonstrate the potential of ZA fibers to provide an environment conducive to cell growth and tissue development.

To minimize costs and time in the production of cultivated meat, various process technologies including cell lines, culture media, and scaffolds are necessary^45^. Furthermore, achieving cultivated meat that closely resembles real muscles instead of minced patties, simulating the alignment technique of muscle cells remains challenging. This study proposes a method that efficiently and simply expresses muscle cell alignment using only physical stimulation, contributing to cost and time savings. We investigated the potential of aligning muscle cells by applying physical stretching stimulation using the excellent ductility of zein. As the strain ratio applied to ZA fiber bundles increased (0–75%), cells became more densely arranged along the fiber direction, closely mimicking the natural muscle alignment (Fig. 6). This approach addresses a significant challenge in the mass production of cultivated meat, as previous techniques were limited to aligning individual or small numbers of strands^14^. To evaluate the effect of strain on myotube differentiation and maturation, C2C12 cells were cultivated on ZA fibers subjected to different strain ratios. Higher strain rates correlated with increased myotube length and a higher proportion of desmin-positive cells, a marker for myogenic differentiation (Fig. 7). Gene expression analysis further supported the progression of myogenic differentiation over time. These findings emphasize the potential of ZA fibers to enhance muscle cell development and maturation, which are important for generating meat-like tissues.

In addition to the simulation of muscle tissue, this study simulates the assembly of muscle, fat, and vessel fibers, mimicking the composition of real meat^38^. Each component exhibited characteristics representative of muscle fibers with aligned myotubes, fat fibers containing lipid droplets, and vessel fibers expressing VE-cadherin (Fig. 8a–c). The ability to assemble these components offers a promising approach to mimic the composition of real meat. Furthermore, the maintenance of fiber structure even when the cultivated meat is pan-fried suggests the possibility of creating textured meat products (Fig. 8d–f).

In conclusion, this study demonstrates the cost-effective and straightforward production of fiber scaffolds using plant-based materials, eliminating the need for toxic chemicals. The utilization of plant-based materials, especially zein and alginate, offers a sustainable alternative for the traditional use of animal-derived materials. However, the challenge of mitigating the distinctive aroma and flavor associated with zein could pose a hurdle for the broader acceptance of cultivated meat. Further investigation is necessary to develop effective techniques capable of diminishing or eliminating these sensory characteristics, which significantly impact the taste and consumer acceptance of cultivated meat products. Moreover, challenges related to scalability, cost-effectiveness, and variations in material properties need careful consideration. Continuous research is important for refining and optimizing the techniques used in this study, especially focusing on addressing potential fluctuations in the quality and characteristics of plant-derived materials. Through further research and development, we believe that our approach will provide the foundation for a field of sustainable and ethical meat alternatives.

## Methods

### Reagents

Sodium alginate was purchased from Junsei Chemical Co. (Tokyo, Japan). A live/dead cell viability kit was purchased from Invitrogen (Carlsbad, CA, USA). Tartrazine was purchased from GreenTech (Daejeon, South Korea). Horse serum and fungizone were purchased from Gibco (Grand Island, NY, USA). Dulbecco’s Modified Eagle’s medium (DMEM), penicillin-streptomycin (P/S), fetal bovine serum (FBS), L-glutamine, and phosphate-buffered saline (PBS; pH 7.4) were purchased from WelGene (Daegu, Gyeongbuk, South Korea). Zein powder, calcium chloride dihydrate, AESO, PEGDA (molecular weight: 575 g/mol), lithium phenyl-2,4,6-trimethylbenzoylphosphinate (LAP, ≥95%), collagenase I, entactin-collagen-laminin (ECL), insulin, linoleic acid, Nile red, bovine serum albumin, and Triton X-100 were purchased from Sigma-Aldrich (St. Louis, MO, USA). All other chemicals used in this study were of analytical grade.

### Zein-coated alginate fiber preparation

The zein-coated ink was prepared by solubilizing zein in 70% ethanol containing 5% CaCl_2_ aqueous solution. The ZA fibers were prepared using a wet-spinning technique (Fig. 1). Briefly, sodium alginate was dissolved in PBS (1% w/v) and prepared in a 10 mL syringe. After the zein-coated ink was placed in a coagulation bath, alginate was injected into the bath at a rate of 0.05 mL/min using a syringe pump (Legato 101; KD Scientific, Holliston, MA, USA). The ZA fibers were collected on the take-up roller and washed several times with PBS to remove residual ethanol and Ca^2+^.

### Mechanical measurements

A CT3 Texture Analyzer (Brookfield, Toronto, ON, Canada) with a 4500 g load cell (Brookfield, Toronto, ON, Canada) in tensile mode was used to measure the mechanical properties of A and ZA fibers. To determine the difference in stress/strain, the fibers were prepared in four states (A, soaked A, ZA, and soaked ZA). The A and ZA fibers were prepared and investigated after 1 h. Soaked A and ZA fibers were prepared and immersed in PBS for 48 h. The samples were cut to a length of 20 mm, and the crosshead velocity was kept at 0.1 mm/s in the tensile measurement. The elastic modulus of the fiber was calculated from the slope over a strain ratio of 5–15%.

### Fabrication of the 3D printed stretching device

Three dimensional printing was performed as previously described^46^. A mixture of AESO/PEGDA (ratio 80:20), 0.5% (w/v) LAP, and 1% (v/v) tartrazine pigment was used as a bioink for the 3D printing of the stretching device^34^. The stretching device model was created using Fusion 360 software (Autodesk, San Rafael, CA, USA) and printed using an IM2 DLP 3D printer (Carima, Seoul, Korea). After 3D printing, the stretching devices were washed several times with PBS prior to UV sterilization.

### Scanning electron microscopy

A and ZA fibers were frozen in a deep freezer at –70 °C for 24 h and lyophilized for 24 h to prevent structural collapse. After lyophilization, the samples were secured to a stub using carbon tape and coated with platinum. Superficial crust morphologies were imaged using an SU-8010 Scanning Electron Microscope (Hitachi, Tokyo, Japan).

### Cell culture and differentiation

C2C12 cells were propagated in DMEM supplemented with 10% heat-inactivated FBS and 1% P/S. The cells were maintained in a humidified atmosphere containing 5% CO_2_ at 37 °C. For muscle fibers, C2C12 cells were seeded onto the fibers at 2 × 10^6^ cells/mL. To induce myogenic differentiation, the medium was replaced with myogenic differentiation medium (MDM; DMEM supplemented with 2% horse serum and 1% P/S), and the cells were cultured for 6 days. MDM was refreshed every 2 days.

### Primary cell isolation, culture, and differentiation

The isolation, cell culture, and differentiation of primary BSCs and adipocytes were performed as previously described^40,47^. Muscle and adipose tissue samples were obtained from Hanwoo cattle at Gyeongbuk Livestock Research Institute. Ethics approval was not required for the acquisition of muscle and fat samples from autopsy of cattle donated for educational purposes. Research of Gyeongbuk Livestock Research Institute is approved by the Ministry of Agriculture, Food and Rural Affairs to handle animal materials. A 0.5 cm biopsy punch was used to isolate a piece of muscle from the longissimus of a 3-year-old cow to obtain primary BSCs. Additionally, primary adipocytes were isolated from 50 g peri-renal fat tissue obtained during an autopsy performed on a 4-year-old cow for anatomical education at University Animal Hospital (Seoul National University, Seoul, South Korea). For primary satellite cells, muscle tissue was immersed in 70% ethanol for 5 min for disinfection and washed with PBS. The fat and connective tissues were carefully removed using forceps and scissors. The tissue was minced and digested with 0.25% collagenase I for 1 h at 37 °C with shaking. The tissue was further digested with 0.25% trypsin/EDTA for 25 min at 37 °C with shaking. The samples were centrifuged for 10 min at 200 × *g*, and the supernatant was discarded. To remove fibroblasts, cell pellets were incubated in uncoated culture flasks for 1 h at 37 °C in a 5% CO_2_ incubator in DMEM supplemented with 10% FBS, 1% P/S, and 250 μg/mL fungizone. Non-adhering primary satellite cells were collected and transferred to cell culture flasks coated with 1 mg/mL ECL. When the cells reached 70% confluence, the growth medium was changed every 2 days, and the cells were passaged at 70% confluence. Primary satellite cells were differentiated or cryopreserved at passage 0 using a CELLBANKER 1 (ZENOAQ, Fukushima, Japan) for subsequent investigation. For myogenic differentiation, primary satellite cells were cultured until 90% confluence, and the medium was replaced with MDM. The cells were then cultured for 6 days, and MDM was refreshed every 2 days.

For primary adipocytes, the adipose tissue was first immersed in 70% ethanol for 5 min for disinfection and washed with PBS. The blood vessels and connective tissues were carefully removed using forceps and scissors. The adipose tissue was then minced and digested with 0.25% collagenase I for 1 h at 37 °C with shaking. The cell suspension was filtered through a 40 μm cell strainer and centrifuged for 5 min at 450 × *g* to obtain cell pellets. The preadipocytes were grown in growth medium (DMEM supplemented with 10% horse serum and 1% P/S). The growth medium was changed every 2 days, and the cells were passaged at 70% confluence. Preadipocytes were differentiated or cryopreserved at passage 0 using a CELLBANKER 1 for subsequent investigation. For adipogenic differentiation, preadipocytes were cultured, and the medium was replaced with adipogenic differentiation medium (ADM; DMEM supplemented with 10% FBS, 1% P/S, 10 μg/mL insulin, and 300 μM linoleic acid). The cells were then incubated, and ADM was refreshed every 2 days.

### Cell viability assay

To investigate the cytotoxicity of ZA fibers compared with that of 2D culture dishes and A fibers, cell viability was investigated using a live/dead cell viability kit containing Calcein–AM and ethidium homodimer-1. Cells were seeded at 2 × 10^6^ cells/mL on 2D culture dishes, A fibers, and ZA fibers, respectively, and incubated for 6 days in a humidified atmosphere of 5% CO_2_ at 37 °C. After 1, 3, and 6 days of incubation, the samples were stained with 500 μL of staining reagent according to the manufacturer’s protocol. Samples were imaged using a Lionheart FX automated microscope (BioTek, Winooski, VT, USA). Cell viability was calculated as the ratio of live cells to the total number of cells.

### Immunocytochemistry

For immunostaining, differentiated cell-laden fibers or cells were fixed with 4% paraformaldehyde at room temperature for 15 min and washed thrice with PBS. For permeabilization, the differentiated cell-laden fibers or cells were incubated with 0.25% Triton X-100 in PBS for 15 min. Then, the samples were blocked in 5% bovine serum albumin in 0.05% Triton X-100 (prepared in PBS) for 1 h and treated with an anti-α-tubulin primary antibody (1:100 in PBS; Developmental Studies Hybridoma Bank, Iowa City, MA, USA), desmin primary antibody (1:200 in PBS; ABclonal, Woburn, MA, USA) or VE-cadherin (1:200 in PBS; Abcam, Cambridge, MA, USA) at room temperature for 1 h. Subsequently, the samples were washed with PBS and stained with Alexa Fluor 488, 555, or 594-labeled secondary antibodies (1:300 in PBS; Invitrogen, Carlsbad, CA, USA) for 1 h and counterstained with 4,6-diamidino-2-phenylindole dihydrochloride (DAPI) (300 nM; Invitrogen, Carlsbad, CA, USA). The samples were visualized using a Lionheart FX automated microscope.

### Quantification of myoblasts/myotubes structure characteristics

Fluorescence images of myoblasts and myotubes were analyzed using Fiji/ImageJ software (National Institutes of Health, Bethesda, MD, USA). Fluorescence was quantified by analyzing the area, circularity, and length of the myoblasts/myotubes corresponding to the short and long axes of the ellipse. The alignment angles of the cells were categorized from −90° to 90° in 10° increments.

### Reverse transcription-quantitative polymerase chain reaction (RT-qPCR)

Total RNA was isolated from C2C12 cells or primary adipocytes using the TRIzol reagent (iNtRON, Seongnam, Gyeonggi, Korea) according to the manufacturer’s instructions. RNA quality was determined at an absorbance of 260 nm (A260) using a BioTek plate reader and Take3 plate reader (BioTek, Winooski, VT, USA). cDNA was synthesized from the total RNA using HKGscript™ 5X RT Premix (HK Genomics, Daejeon, Korea) at 50 °C for 15 min and 70 °C for 15 min. RT-qPCR was performed on a LightCycler^®^ 96 system (Roche, Basel, Switzerland) using the SYBR Green qPCR Master Mix (Genetbio, Daejeon, Korea). The primer sequences are listed in Table 1. Thermal cycling conditions were: 95 °C for 10 min, followed by 40 cycles at 95 °C for 15 s, and 60 °C for 40 s.

**Table 1:** Primer sequences used in real-time RT-qPCR analysis

Gene	Primer sequence (5ʹ–3ʹ)
Mouse *GAPDH*	Forward: CAGTATGACTCCACTCACGGC
	Reverse: CTCGCTCCTGGAAGATGGT
Mouse *Pax7*	Forward: GGGCTCAGATGTGGAATCAG
	Reverse: GGGTAGTGGGTCCTCTCAAA
Mouse *MyoD*	Forward: CTGCTCTGATGGCATGATGGAT
	Reverse: CCTCACTGTAGTAGGCGGTGT
Mouse *MyoG*	Forward: GGCATGTAAGGTGTGTAAGAGG
	Reverse: CTTGAGCCTGCGCTTCTCCCTC
Mouse *MYH1*	Forward: GGCCAGACTGTGCAGCAGGTGT
	Reverse: GGTGACCATCCACAGGAACATC
Bovine *GAPDH*	Forward: CAGTATGATTCCACCCACGGC
	Reverse: ATCTCGCTCCTGGAAGATGGTG
Bovine *PPARγ*	Forward: GAGGACATTCCGTTCCCAAG
	Reverse: TGCACTTTGGTACTCTTGGAGC
Bovine *CEBPα*	Forward: CCGTGGACAAGAACAGCAAC
	Reverse: CGGTCATTGTCACTGGTCAG

### Nile red staining

For lipid droplet staining, the differentiated adipocyte-laden fibers were fixed with 4% paraformaldehyde at room temperature for 15 min and washed thrice with PBS. Samples were incubated for 30 min at 1:1,000 dilution in PBS containing 1 mg/mL Nile red. Samples were then washed thrice with PBS, and the cell nuclei were stained with DAPI. Samples were visualized using a Lionheart FX automated microscope.

### Statistical analysis

Data are presented as means ± standard deviation (s.d.), and means were compared using one-way analysis of variance (ANOVA) followed by Tukey’s test or a two-tailed paired and unpaired Student’s *t* test. Statistical significance was set at *P* < 0.05. All analyses were performed using GraphPad Prism 8.0.2 (GraphPad Software, La Jolla, CA, USA).

### Reporting summary

Further information on research design is available in the Nature Research Reporting Summary linked to this article.

### Supplementary information

Reporting summary

Supplementary_Material_Video_1

Supplementary_Material_Video_2

## Data Availability

The authors declare that all data supporting the findings of this study are available in the paper.
